# To what degree are health insurance enrollees aware of the restrictive conditions of their policies?

**DOI:** 10.1093/eurpub/ckac129.566

**Published:** 2022-10-25

**Authors:** FJP Van der Hulst, L Holst, AEM Brabers, JD De Jong

**Affiliations:** 1 NIVEL, Utrecht, Netherlands; 2 Maastricht University, Maastricht, Netherlands

## Abstract

**Background:**

Over recent decades, many Western countries have added market incentives to their health systems. In a system of managed competition, health insurers are supposed to be prudent purchasers of health care on behalf of their enrollees. They can contract healthcare providers selectively. Enrollees who choose a health insurance policy with restrictive conditions, will have to make a co-payment if they consult a non-contracted provider. If the co-payment is unexpected, it may cause a problem for the enrollee. This study aims to gain insight into enrollees’ awareness of the conditions of such health insurance policies in the Netherlands.

**Methods:**

In August 2020, an online questionnaire was sent out via health insurers to enrollees with restrictive health plans. In total 13,588 enrollees responded. Descriptive statistics and logistic regression analyses were performed on various outcome measures relating to enrollees’ awareness of the restrictive conditions.

**Results:**

One fifth (19%) of the respondents appeared to be totally unfamiliar with the policy conditions. Men, younger people, people with a low level of education, a lower income, a poorer health status, and non-care users were found to be less familiar with the conditions (p = 0.00 for all). 62% who wanted to visit a healthcare provider whose care was not fully reimbursed, still went to that provider. Of those who had to pay extra because hospital care was not fully reimbursed, 62% did not know this in advance and 30% indicated that paying extra was a serious problem.

**Conclusions:**

Not all enrollees who choose a policy with restrictive conditions are aware of the consequences of receiving care from a non-contracted provider. There seems room for improving the information provision, in particular for people with a low income and people with a poorer health status, as these groups more often reported unawareness about having to pay extra and more often faced financial problems.

**Key messages:**

• Not all enrollees are well informed about their policy with restrictive conditions.

• Selective contracting does not always affect enrollees’ choice of a healthcare provider.

